# Structure of Coagulation Factor II: Molecular Mechanism of Thrombin Generation and Development of Next-Generation Anticoagulants

**DOI:** 10.3389/fmed.2018.00281

**Published:** 2018-10-02

**Authors:** Mathivanan Chinnaraj, William Planer, Nicola Pozzi

**Affiliations:** Edward A. Doisy Department of Biochemistry and Molecular Biology, Saint Louis University School of Medicine, St. Louis, MO, United States

**Keywords:** coagulation cascade, prothrombin, thrombin, anticoagulants, X-ray crystallography, single-molecule FRET, drug discovery

## Abstract

Coagulation factor II, or prothrombin, is a multi-domain glycoprotein that is essential for life and a key target of anticoagulant therapy. In plasma, prothrombin circulates in two forms at equilibrium, “closed” (~80%) and “open” (~20%), brokered by the flexibility of the linker regions. Its structure remained elusive until recently when our laboratory solved the first X-ray crystal structure of the zymogen locked in the predominant closed form. Because of this technical breakthrough, fascinating aspects of the biology of prothrombin have started to become apparent, and with this, novel and important questions arise. Here, we examine the significance of the “closed”/“open” equilibrium in the context of the mechanism of thrombin generation. Further, we discuss the potential translational opportunities for the development of next-generation anticoagulants that arise from this discovery. By providing a structural overview of each alternative conformation, this minireview also offers a relevant example of modern structural biology and establishes a practical workflow to elucidate the structural features of analogous clotting and complement factors.

## Introduction

In response to vascular injury, coagulation factor II (FII), or prothrombin, is converted to its active form thrombin by prothrombinase, a macromolecular complex composed of factor Xa (fXa), factor Va (fVa), calcium ions, and phospholipids (1). Once in the circulation, thrombin converts fibrinogen into fibrin, activates platelets and increases endothelial permeability thereby halting the loss of blood at the site of injury (2–4) and facilitating vascular remodeling. Because of this critical role in biology, the prothrombin/thrombin axis remains an attractive target for anticoagulant therapy (5, 6).

## Prothrombin gene and domain organization

In humans, prothrombin is encoded by the F2 gene, which is located on the short arm of chromosome 11, at position 11.2 (6). The gene contains 14 exons spanning 21 kb, and its structural integrity is critical for life. Mice lacking prothrombin die prematurely at the embryonic stage due to bleeding complications (7). Single-nucleotide polymorphisms (SNPs) found in patients are often associated with moderate to severe bleeding phenotypes and the mutation G20210A in the 3′ untranslated region of the F2 gene is a well-established risk factor for thrombophilia (8).

Prothrombin is synthesized by the hepatocytes in the liver as a single pre/pro-polypeptide composed of 622 amino acids (5), although elevated mRNA levels have also been detected in neurons and glia cells suggesting important yet unexplored roles of this protein in the central nervous system (9). Before secretion into the plasma, prothrombin undergoes extensive post-translational modifications including removal of the pre/pro peptide at the N-terminus (43 amino acids), addition of three N-glycosylations at positions 78, 100, and 373 and conversion of the first 10 residues of glutamic acid (Glu) to γ-carboxy glutamic acid (Gla) (5, 10). The presence of glycans at position 373 increases the thermodynamic stability of the protein and confers protection to proteolysis without affecting the catalytic activity of thrombin (11). The role of the other two N-glycosylations at positions 78 and 100 remains unclear. The 10 Gla residues provide a calcium-dependent anchoring point to negatively charged phospholipid. Since prothrombin conversion to thrombin occurs on the membranes, pharmacological inhibition of the γ-carboxyl transfer reaction in the liver by vitamin K analogs, such as warfarin, represents an effective and widespread strategy to achieve profound anticoagulation in clinical practice (12). Likewise, mutations Glu7→ Lys in prothrombin Nijmegen and Glu29→ Gly in prothrombin Shanghai are associated with a severe bleeding diathesis (8).

**Figure 1:**
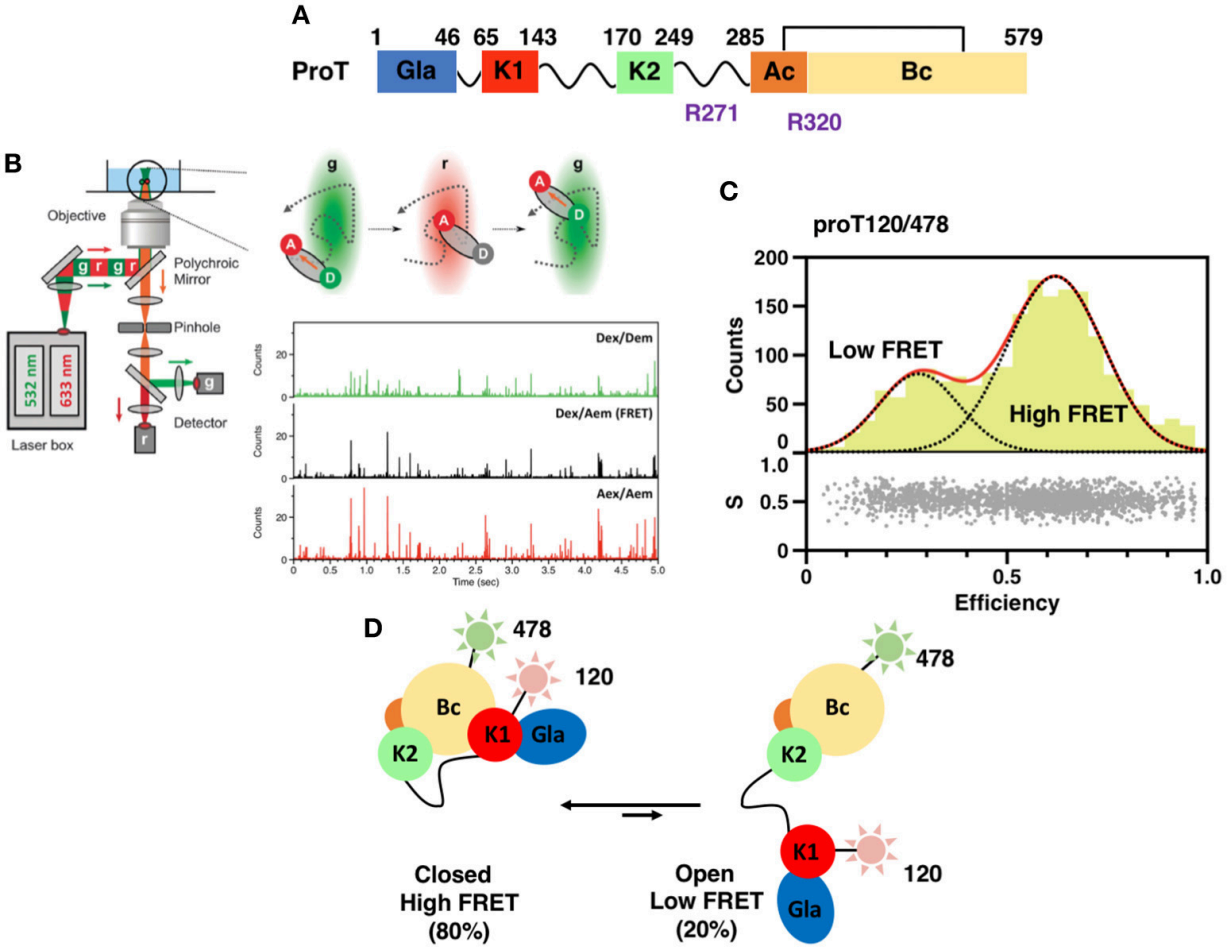
**(A)** Color-coded domain architecture of prothrombin displaying the location of proteolytic sites R271 and R320 that are cleaved by the prothrombinase complex. **(B)** Scheme of a confocal microscope equipped for sm-PIE-FRET experiments. Briefly, the donor and acceptor dyes are excited with a ps pulsed diode laser at 532 and 633 nm, respectively. To achieve PIE, the 532 nm laser is electronically delayed (25–50 ns) relative to the 633 nm laser. Signals from single molecules are observed as bursts of fluorescence and detected with two SPAD detectors. Data are stored in the Time-tagged Time-resolved Mode (15, 16). **(C)** smFRET histogram for the prothrombin mutant S120C/S478C (proT120/478) in which fluorescent dyes were attached at position 120 in kringle 1 and 478 in the B-chain (16). The bottom section of the graph depicts the stoichiometry, S, vs. FRET efficiency for each diffusing molecule. The upper section shows the one-dimensional efficiency histogram of the molecules in the bottom section. ProT120/478 shows two distributions of molecules with distinct FRET efficiencies (low and high), supportive of the existence of closed and open conformations in solution. **(D)** Schematic models of closed and open conformations built from smFRET measurements.

The mature form of prothrombin circulates in the plasma at a concentration of 0.1 mg/ml and has a half-life of about 60 h (13, 14). It contains four domains connected by three intervening linkers, Lnk1 (residues 47–64), Lnk2 (residues 144–169), and Lnk3 (residues 249–284) totaling 579 amino acids (Figure 1A) (15). The N-terminal Gla-domain (residues 1–46), named after the posttranslational modifications, is followed by two kringles, kringle-1 (residues 65–143) and kringle-2 (residues 170–248), and a canonical protease domain (residues 285–579). The protease domain contains the A chain (residues 285–320) and the B chain (residues 321–579) which are connected by a conserved disulfide bond (Cys293–Cys439). The catalytic triad (His363, Asp419, and Ser525) is hosted in the B-chain and strategically located in a deep pocket surrounded by flexible loops that control access to and steer substrates toward the active site (17).

Kringles are small structural elements (i.e., 60 amino acids) found in hundreds of proteins in the genome and usually mediate protein-protein interactions (18). In agreement with this knowledge, previous studies have proposed interactions between kringle-1 and kringle-2 with fVa and fXa during the assembly of the prothrombinase complex (19, 20). Targeting kringles could, therefore, provide a rational strategy to develop anticoagulants. Interestingly, kringle-2 was found to be the locale of autoantibodies in the antiphospholipid syndrome in patients with Systemic Lupus Erythematosus (21) and was also found to act as a potential neuroinflammatory factor that stimulates microglial toll-like receptor 4 (TLR4) (22). Since kringle-2 is part of prothrombin fragment 1.2 which is released from prothrombin upon activation (23), neutralization or enhanced clearance of this fragment from the circulation may be beneficial to patients with chronic inflammation.

## Structures of the closed and open forms of prothrombin

Crystallization of zymogens is problematic compared to the analogous proteases because of the complex modular assembly, higher molecular flexibility, and autoactivation (24–27). To overcome this limitation and obtain structural insights into the mechanisms of zymogen to protease conversion, single-molecule Förster Resonance Energy Transfer (smFRET) has recently emerged as a reliable and affordable tool for structural biologists. By recording the energy that is transferred from an excited molecule (Donor) to a second molecule with spectral overlap (Acceptor) at the single molecule level, smFRET measures intra-molecular distances on nanometer scale with high precision (28–30), detects subpopulation of molecules and monitors kinetics of interconversions (31, 32) (Figure 1B). This information can be used to generate low-resolution structural models of the protein of interest by triangulation and molecular modeling (15, 16, 33), thus providing the necessary structural foundations to design new optimized reagents suitable for crystallization. Measurements are typically carried out on a confocal microscope equipped with Pulsed Interleaved Excitation (PIE) and single-photon avalanche detectors (SPAD) to sort populations of single molecules according to stoichiometry (S) and FRET efficiency (E) (Figure 1C) (34, 35).

**Figure 2:**
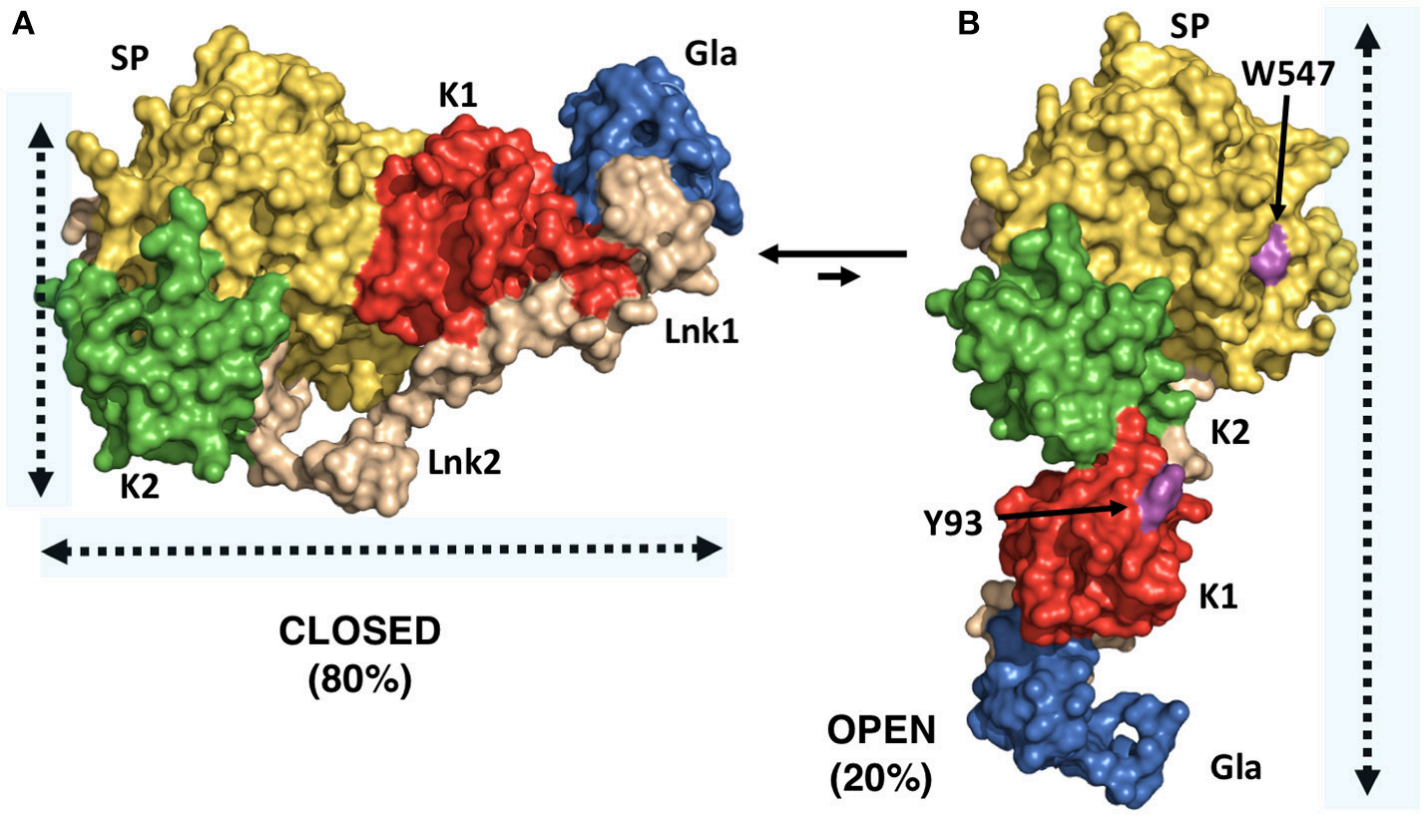
X-ray crystal structures of closed and open conformations of prothrombin. **(A)** Structure of the prothrombin mutant S101C/A470C (proTCC) solved at 4.0Å resolution (PDB ID: 6C2W) (15). **(B)** Structure of the prothrombin mutant Δ154–167 (proTΔ154–167) solved at 2.2Å resolution (PDB ID: 5EDM) (33). Key residues Trp547 in the protease domain and Tyr93 in the kringle-1 are shown in magenta.

A successful example of the above-mentioned workflow is provided by the structural studies of the clotting factor II. A total of 8 prothrombin FRET pairs were engineered by substituting serine residues with cysteine to introduce fluorescent dyes via maleimide chemistry (15, 16). Analysis of the FRET histograms revealed that prothrombin exists in two forms at equilibrium, closed (~80%) and open (~20%) (Figure 1C) [32], and enabled the generation of structural models for each alternative conformation (Figure 1D). This led to “educated” protein engineering experiments. Mutation of Tyr93 to Ala, Trp547 to Ala, or deletion of >15 amino acids from Lnk2 shifted the equilibrium toward the open form by disrupting the intramolecular interaction between kringle-1 and the serine protease domain. In contrast, engineering of an artificial disulfide bond between residue 101 in kringle-1 and residue 470 in the serine protease domain resulted in stabilization of the closed form. Importantly, locking the conformational ensemble in the closed or open forms produced crystallization under several conditions. The X-ray crystal structure of the double mutant proTS101C/A470C (proTCC, closed, PDB ID: 6C2W) was solved at 4.1Å resolution (Figure 2A) (15). The X-ray crystal structure of the deletion mutant 154–167 (proTΔ154–167, open, PDB ID: 5EDM) was solved at 2.2Å (Figure 2B) (33).

The difference between “closed” and “open” conformations of prothrombin is striking and best captured by the video uploaded in the Supplementary Material. Prothrombin transitions from an “L-closed” to and “I-open” shape due to the relocation of fragment-1 away from the protease domain. In the closed form, kringle-1 sits on top of the catalytic pocket, therefore, blocking access of substrates to the active site, as well as hiding portions of the flexible autolysis loop. Given that prothrombin undergoes autoactivation when histones are dumped in the circulation upon cellular damage (24, 36), and that the autolysis loop is targeted by bacterial proteases that generate thrombin bypassing the canonical activation pathway (37), the closed conformation of prothrombin may represent an autoinhibited, proteolytically resistant form of the zymogen in the circulation with improved plasma half-life. This protection mechanism may be shared with other plasma proteins such as factor XII, which has been recently shown to have single-chain catalytic activity (26). In contrast, the open form is elongated, the active site unshielded by kringle-1 and overall, it exposes an additional surface area of ~1253 Å (2), mostly hydrophobic. This form might, therefore, interact with specific membrane receptors via kringle-1, dimerize and have different proteolytic susceptibly compared to the closed form.

## Thrombin generation

Activation of prothrombin by the prothrombinase complex entails cleavage at two distinct sites, Arg271 and Arg320, along two alternative pathways that generate the inactive intermediate prethrombin-2 or the enzyme meizothrombin, respectively (1). It remains unclear how prothrombin interacts with the prothrombinase complex at the molecular level and what are the structural determinants enabling the selection of the pathway of activation. Krishnaswamy's group was the first to propose a model of prothrombin activation whereby the zymogen binds to a single pre-existing conformation of the prothrombinase complex favoring interaction of Arg320 with the active site of factor Xa (38). They also proposed that the ordered cleavage at Arg320 followed by Arg271 is driven by ratcheting of prothrombin due to a structural reorganization of the active site (39). In another study, Nesheim's group challenged this model proposing the existence of two interconverting forms of prothrombinase at equilibrium responsible for cleaving at Arg271 and Arg320 (40, 41). The partition between these two conformations would then dictate the selection of the pathway. In our view, these models are not mutually exclusive. They suggest that conformational changes occur in both enzyme and substrate at the same time. We have recently discovered that closed and open conformations of prothrombin are substrates of the prothrombinase complex, yet their activation follows distinct pathways (15). The closed form is cleaved first at Arg320 to form the active intermediate meizothrombin whereas the open form is cleaved first at Arg271 to generate the inactive intermediate prethrombin-2. These results resonate with the structural flexibility of the substrate inferred by Krishnaswamy's group and propose a new mechanistic framework envisioning a long-range communication between the conformations of the zymogen and the presentation of the cleavage sites. Additionally, given that proTCC is covalently stabilized in the closed form and only minor adjustments would be possible for proTCC upon binding to the prothrombinase complex, our results also vouch for the existence of multiple conformations of fXa to accommodate the structural diversity of prothrombin. Data from Brufatto and Nesheim (40), Kim and Nesheim (41), Srivasatava et al. (42), and Qureshi et al. (43) reporting flexibility of fXa upon binding to the lipids and fVa support our hypothesis. And so do recent structural studies from Pomowski et al. (44), Lechtenberg et al. (45) and Lee et al. (46). Rigid-body docking of the closed form of prothrombin to the available structure of the prothrombinase complex returns unreasonable models of the tertiary complex, thus suggesting that the orientation of fXa bound to fVa may be different compared to what depicted in the crystal structure.

In light of these new developments, we propose that binding of prothrombin to prothrombinase occurs primarily in the closed form, which is the most abundant conformation in solution (i.e., 80%). Cleavage of prothrombin at Arg320 switches to the intermediate meizothrombin in the open form that is then cleaved at Arg271 to produce the mature enzyme thrombin. In solution, without constraints, cleavage of Arg320 promotes the complete opening of the prothrombin structure through a jack-knife mechanism, as observed in meizothrombin (15). However, in the context of the prothrombinase complex, the structure likely remains compressed because of fVa. Twisting of the protease domain and kringle-2 on top of fragment-1 that remains anchored to the membranes (47–49) and elongation of fXa may explain how Arg271 is relocated to the active site of fXa. Future studies are needed to test this hypothesis and provide a clearer mechanistic understanding of such an essential and paradigmatic reaction of blood coagulation.

## Development of next-generation anticoagulants

The recent advent of direct oral anticoagulants (DOACs) has marked the most significant change for the long-term management of patients at high risk of thrombosis. DOACs offer a rapid onset and offset of action with more predictable dose response and pharmacodynamics, display a wider therapeutic window and have less food-drug and drug-drug interactions compared to traditional vitamin K antagonists, thereby allowing physicians to reduce monitoring and improve compliance (50–52). Yet, these drugs are far from ideal and their widespread adoption is still limited by high costs, lack of antidotes, and in particular by gastrointestinal and intracranial bleeding side effects. While the price of these medications is a matter of ethical debates, the pharmacological side effects are tightly linked to their mechanism of action which relies on the blockade of the catalytic activity of key procoagulant enzymes, i.e., factor Xa and thrombin.

The emerging new structural framework of prothrombin could lead to the development of next-generation anticoagulants with a novel mechanism of action and a safer pharmacological profile. Guided by the structures of closed and open conformations of prothrombin, it is now possible to design compounds that would fit into the active site pocket of the zymogen with the goal of competing with Tyr93 and select ligands from phage display or antibody libraries that would electively recognize the open conformation of the prothrombin. These (bio)molecules are expected to stabilize the open form, force the activation pathway through prethrombin-2 (39, 53) and work as anticoagulants by limiting rather than blocking thrombin activity. And while this pharmacological strategy awaits validation, it is interesting to note that the naturally occurring mutations Ala362Thr, Glu466Ala, and Gly548Ala which are associated with a mild bleeding phenotype cluster in the proximity of the interface between kringle-1 and the serine protease domain and may perturb the closed-open conformational equilibrium (8). Likewise, the mutation Glu157Lys in prothrombin Canberra occurs in Lnk2, which is a key structural element of the conformational equilibrium.

## Conclusions

Mounting evidence indicates that clotting factor II, or prothrombin, is a flexible protein and such flexibility may be the key to accomplishing its physiological function. This observation echoes recent structural findings on other plasma proteins such as plasminogen (54), ADAMTS-13 (55), factor XII (26), and β_2_-glycoprotein I (56), which have been shown to adopt alternative conformational states that correlate with their pathophysiological function. Such a discovery may also explain as to why crystallization of prothrombin, and zymogens in general, has been remarkably challenging for years and warn investigators from interpreting functional aspects of multi-domain flexible proteins from a single structural snapshot.

Integrating single-molecule spectroscopy with protein engineering and X-ray crystallography is an emerging universal workflow that enables identification of subpopulations of molecules, characterization of their structural features and investigation of their functional role. This approach is therefore poised for successes as it provides mechanistic insights into fundamental processes of biology and offers rational strategies for the development of novel therapeutics aimed at locking “active” or “inactive” protein conformational states.

## Author contributions

All authors listed have made a substantial, direct, and intellectual contribution to the work, and approved it for publication.

### Conflict of interest statement

The authors declare that the research was conducted in the absence of any commercial or financial relationships that could be construed as a potential conflict of interest.

## Supplementary material

Supplementary Video 1 Pymol video generated after superposition of the serine protease domain and kringle-2 of the closed (PDB ID: 6C2W) (15) and open (PDB ID: 5EDM) (33) conformations of prothrombin.
